# Supplementary material for: Mitochondrial Variation of Bottlenose Dolphins (Tursiops truncatus) from the Canary Islands Suggests a Key Population for Conservation with High Connectivity within the North-East Atlantic Ocean
Source: Animals (Basel). 2024 Mar 14;14(6):901. doi: 10.3390/ani14060901 (PMC10967437; doi:10.3390/ani14060901)
Supplement: Supplementary file 1 [file animals-14-00901-s001.zip › animals-2878497-supplementary.pdf]

Supplemental Materials

Mitochondrial Variation of Bottlenose Dolphins (*Tursiops truncatus*) from the Canary Islands Suggests a Key Population for Conservation with High Connectivity within the North-East Atlantic Ocean

Daniel A. Gómez-Lobo [1,2], Agustín P. Monteoliva [2], Antonio Fernandez [3], Manuel Arbelo [3], Jesús de la Fuente [3], Mónica Pérez-Gil [4], Nuria Varo-Cruz [4], Antonella Servidio [4], Enrique Pérez-Gil [4], Yaisel J. Borrell [1] and Laura Miralles [1,2,*]

[1] Department of Functional Biology, University of Oviedo, 33006 Oviedo, Spain; dagomezlobo@uc.cl (D.A.G.-L.); borrellyaisel@uniovi.es (Y.J.B.)
[2] Department of Environmental Genetics, Ecohydros, 39600 Maliaño, Spain; apmonteoliva@ecohydros.com
[3] Veterinary Histology and Pathology, Atlantic Center for Cetacean Research, Institute of Animal Health and Food Safety (IUSA), Veterinary School, University of Las Palmas de Gran Canaria, 35001 Las Palmas, Spain; antonio.fernandez@ulpgc.es (A.F.); manuel.arbelo@ulpgc.es (M.A.); jesus.delafuente@ulpgc.es (J.d.l.F.)
[4] Cetaceans and Marine Research Institute of the Canary Islands (CEAMAR), 35509 Las Palmas, Spain; monica@ceamar.org (M.P.-G.); nuriavaro@hotmail.com (N.V.-C.); antonella@ceamar.org (A.S.); kikelanza@gmail.com (E.P.-G.)
* Correspondence: miralleslaura@uniovi.es

Table S1. Detailed sampling data of bottlenose dolphins from the Canary Islands. New haplotypes from this study are marked in bold. New haplotype numeration follows date of sampling (CAN1 is the oldest and CAN15 the most recent to date)

N	Sample	Type	Date	Locality	Haplotype	Accesion Number
**1**	**CET0296**	**Stranding**	**11/5/05**	**Gran Canaria**	**CAN1**	**OQ656769**
**2**	**CET0635**	**Stranding**	**18/11/12**	**Gran Canaria**	**CAN4**	**OQ656772**
3	CET0724	Stranding	23/7/14	Gran Canaria	Ttrunc12	KF650794
4	CET1200	Stranding	4/11/21	Gran Canaria	Ttrunc45	KF650827
**5**	**CET0311**	**Stranding**	**18/9/05**	**Fuerteventura**	**CAN2**	**OQ656770**
6	CET1133	Stranding	31/10/20	Fuerteventura	Ttrunc46	KF650828

**7**	**CET0564**	**Stranding**	**22/3/11**	**Lanzarote**	**CAN3**	**OQ656771**
8	CET0305	Stranding	18/7/05	Lanzarote	BSEA6	KF570328
9	CET0595	Stranding	3/12/11	Lanzarote	Ttrunc12	KF650794
10	CET0640	Stranding	20/12/12	Lanzarote	Ttrunc49	KF650831
**11**	**CET0662**	**Stranding**	**2/5/13**	**Tenerife**	**CAN5**	**OQ656773**
**12**	**CA_Ttr02_120721**	**Biopsy**	**12/7/21**	**Tenerife**	**CAN5**	**OQ656773**
**13**	**CA_Ttr01_140721**	**Biopsy**	**14/7/21**	**Tenerife**	**CAN6**	**OQ656774**
**14**	**CET1209**	**Stranding**	**31/1/22**	**Tenerife**	**CAN6**	**OQ656774**
**15**	**CA_Ttr04_130422**	**Biopsy**	**13/4/22**	**Tenerife**	**CAN7**	**OQ656775**
**16**	**CA_Ttr01_160422**	**Biopsy**	**16/4/22**	**Tenerife**	**CAN9**	**OQ656777**
**17**	**CA_Ttr02_120422**	**Biopsy**	**12/4/22**	**Tenerife**	**CAN11**	**OQ656779**
**18**	**CA_Ttr02_130422**	**Biopsy**	**13/4/22**	**Tenerife**	**CAN12**	**OQ656780**
**19**	**CA_Ttr03_150422**	**Biopsy**	**15/4/22**	**Tenerife**	**CAN13**	**OQ656781**
**20**	**CA_Ttr05_150422**	**Biopsy**	**15/4/22**	**Tenerife**	**CAN14**	**OQ656782**
**21**	**CA_Ttr03_160422**	**Biopsy**	**16/4/22**	**Tenerife**	**CAN15**	**OQ656783**
22	CA_Ttr02_160422	Biopsy	16/4/22	Tenerife	TT016	DQ073656
23	CA_Ttr02_150422	Biopsy	15/4/22	Tenerife	TT027	DQ073667
24	CET0526	Stranding	29/3/10	Tenerife	Ttrunc2	KF650784
25	CET0407	Stranding	18/1/08	Tenerife	Ttrunc7	KF650789
26	CET0903	Stranding	2/4/18	Tenerife	Ttrunc7	KF650789

27	CA_Ttr01_130422	Biopsy	13/4/22	Tenerife	Ttrunc7	KF650789
28	CET1042	Stranding	23/11/19	Tenerife	Ttrunc9	KF650791
29	CA_Ttr03_130422	Biopsy	13/4/22	Tenerife	Ttrunc9	KF650791
30	CA_Ttr01_120422	Biopsy	12/4/22	Tenerife	Ttrunc11	KF650793
31	CA_Ttr03_120422	Biopsy	12/4/22	Tenerife	Ttrunc11	KF650793
32	CET0450	Stranding	13/5/08	Tenerife	Ttrunc13	KF650795
33	CA_Ttr01_110721	Biopsy	11/7/21	Tenerife	Ttrunc13	KF650795
34	CA_Ttr01_140422	Biopsy	14/4/22	Tenerife	Ttrunc13	KF650795
35	CA_Ttr03_140422	Biopsy	14/4/22	Tenerife	Ttrunc13	KF650795
36	CA_Ttr01_150422	Biopsy	15/4/22	Tenerife	Ttrunc13	KF650795
37	CA_Ttr02_140422	Biopsy	14/4/22	Tenerife	Ttrunc27	KF650809
**38**	**CA_Ttr01_250721**	**Biopsy**	**25/7/21**	**La Gomera**	**CAN7**	**OQ656775**
**39**	**CA_Ttr03_080921**	**Biopsy**	**8/9/21**	**La Gomera**	**CAN7**	**OQ656775**
**40**	**CA_Ttr01_290721**	**Biopsy**	**29/7/21**	**La Gomera**	**CAN8**	**OQ656776**
**41**	**CA_Ttr03_060921**	**Biopsy**	**6/9/21**	**La Gomera**	**CAN9**	**OQ656777**
**42**	**CA_Ttr01_080921**	**Biopsy**	**8/9/21**	**La Gomera**	**CAN10**	**OQ656778**
43	CA_Ttr02_060921	Biopsy	6/9/21	La Gomera	TT016	DQ073656
44	CA_Ttr04_060921	Biopsy	6/9/21	La Gomera	TT016	DQ073656
45	CA_Ttr02_070921	Biopsy	7/9/21	La Gomera	TT027	DQ073667
46	CET0730	Stranding	11/10/14	La Gomera	Ttrunc11	KF650793

47	CA_Ttr02_080921	Biopsy	8/9/21	La Gomera	Ttrunc11	KF650793
48	CA_Ttr01_070921	Biopsy	7/9/21	La Gomera	Ttrunc13	KF650795
49	CA_Ttr01_040921	Biopsy	4/9/21	La Gomera	Ttrunc46	KF650828

Table S2. Comparison of genetic diversity indexes of Biopsies and Stranding samples in bottlenose dolphins from the Canary Islands including sample size (*n*), segregating sites (*S*), number of haplotypes (Nh), number of unique haplotypes (h), haplotype diversity (Hd), nucleotide diversity (π), and average number of nucleotide differences (*k*). SD = standard deviation.

Type	*n*	*S*	Nh	Hd (SD)	π	*k*
Biopsy	31	36	19	0.955 (0.020)	0.01564	9.916
Stranding	18	36	16	0.987 (0.023)	0.01625	10.320
